# Virulence, antimicrobial resistance, and mobile genetic elements profile of *Clostridium perfringens* isolated from animal derived food chains

**DOI:** 10.1016/j.onehlt.2026.101398

**Published:** 2026-03-30

**Authors:** Zhaoyu Chang, Haoyu Zhao, Xinru Wang, Yue Dong, Juan Wang, Deyang Guo, Kailiang Han, Yanfen Jiang

**Affiliations:** College of Veterinary Medicine, Northwest A&F University, 712100 Yangling, Shaanxi, China

**Keywords:** *Clostridium perfringens*, Bioinformatics analysis, Antimicrobial resistance genes (ARGs), Multilocus sequence typing, Mobile genetic elements (MGEs)

## Abstract

*Clostridium perfringens* (*C. perfringens*) is an important opportunistic zoonotic pathogen, ubiquitous in nature, causing food poisoning, enteric disease, and histotoxic infections in humans and animals. Genomic investigation of *C. perfringens* from animal-derived food product chains remains limited. In current study, 33 isolates were analyzed and phylogenetic relationships were elucidated with 119 published genomes, five clusters were classified with predominance of Cluster V. All isolates were classified into 16 STs including 7 novel STs (ST1072-ST1079). Twenty-seven virulence genes with conserved virulence backbone but heterogeneous toxinotype-defining loci (*cpe*, *etx*, *netB*), pCW3, pCP13, pIP404 plasmid families in 54.5% isolates, extensive diversity of ISs, *Clostr_vB_CpeS_CP51*(39.4%) and *phiSM101*(33.3%) with phage-borne toxin genes (*ccp*, *nanH*, *plc*, and *hlyA*) were identified. *tetA(P)* (45.5%)/*tetB(P)* (39.4%)/*erm(Q)* (28.6%) were dominated ARGs, 24.2% of isolates showed multidrug resistance profile while WHD-1 (type G) harbored 8 ARGs. Notably, one *optrA*-positive isolate was recovered from beef slaughterhouse air. Further, 2 isolates carried *cpe* + *IS1151* + *becA* linkage, *nanH*/*nanI*/*nanJ* sialidase genes, accompanying pIP404 but not pCW3 with *IS1469*/*IS1470*, *cpb2* and ARGs absent, is obvious different from characteristic of type F strain. cgMLST offers more accurately resolves fine-scale chromosomal relationship than seven-locus MLST. Together, this study provides an integrated genomic overview of *C. perfringens* from animal-derived food chains, showing that prophage gain/loss, IS expansion, and plasmid carriage vary semi-independently and jointly shape ARGs and virulence profiles. It highlights the need for lineage-resolved genomic surveillance that considers both conserved chromosomal scaffold and dynamic MGE landscape to control strategies for this zoonotic pathogen.

## Highlights

• Seven novel MLST sequence types were identified among animal-derived *C. perfringens* isolates.
• Both *c-cpe* and *p-cpe* isolate shared same *cpe* + *IS1151* + *becA* linkage accompanying pIP404-like plasmid.
• Type F isolates from ovine-origin possessing potential risk of *C. perfringens* food-borne disease.
• cgMLST offers a more refined resolution of fine-scale chromosomal relationships among the isolates compare to MLST.

## 1 Introduction

*Clostridium perfringens* (*C. perfringens*) is a Gram-positive, spore-forming, facultative anaerobe which is widely distributed in nature, particularly in soil, feces, and the gastrointestinal tracts of humans and animals. *C. perfringens* causes numerous enterotoxic and histotoxic illnesses as well as food-borne gastrointestinal (GI) disorders in both humans and animals [1,2]. It has been recognized as one of the primary bacterial pathogens responsible for foodborne disease outbreaks in the United States and several European countries. In the USA, it ranks as the second most common bacteria causing of foodborne illness [3], with an estimated one million cases reported annually. The pathogenicity of *C. perfringens* is largely attributed to its production of approximately 20 exotoxins and enzymes. Thirteen of these toxins—namely α, β1, β2, ε, ι, θ, γ, δ, λ, η, μ, κ, and *ν* have been well-characterized, with α, β, ε, ι, CPE, and NetB recognized as the lethal toxin [1,2,4]. Based on the capacity to secrete those six major toxins, this bacterium is classified into seven toxinotypes (types A ∼ G) [4].

Toxinotyping has served as a cornerstone for epidemiological investigations, pathogenic lineage identification, and clinical diagnostics of *C. perfringens* infections for a long time. However, its resolution is limited when delineating strain-specific virulence profiles or elucidating mechanisms of pathogenesis [5]. To overcome these limitations, multilocus sequence typing (MLST), a high-resolution molecular typing method based on the sequencing of housekeeping genes has been widely applied [6,7]. MLST is a molecular typing method that integrates bioinformatics and high-throughput techniques, and it is regarded as the gold standard for bacterial typing [8]. A study analyzed 8 housekeeping genes of CPE-expressing and non-CPE-expressing *C. perfringens* isolates by using Deguchi's MLST protocol [9], this method has also been employed to examine the population diversity and genetic evolution of *C. perfringens* [6,10]. MLST enables the characterization of clonal lineages and phylogenetic relationships by assigning sequence types (STs) based on allelic variations in loci such as *plc*, *dut*, *glpK*, *gmk*, *sod*, *tpi*, *ddlA*, and *recA*, or alternatively, the PubMLST-adopted scheme involving *gyrB*, *sigK*, *sodA*, *groEL*, *pgk*, *nadA*, *colA*, and *plc* [10].

Multidrug-resistant (MDR) *C. perfringens* strains have been reported increasingly in recent years, particularly in livestock and poultry, one study reported that MDR rates reached 30.6% and up to 74% in foodborne animal-origin isolates, highlighting a rising public health concern [11]. Numerous studies have reported high resistance rates to tetracyclines, macrolides (e.g., erythromycin), lincosamides, aminoglycosides, sulfonamides, and fluoroquinolones in various animal-derived isolates [6,[12], [13], [14]]. Alarmingly, recent discoveries of plasmid-encoded ABC transporter genes such as *optrA* have demonstrated cross-resistance to oxazolidinones and phenicols [15], further complicating treatment strategies.

The dissemination of virulence and resistance genes in *C. perfringens* is s heavily facilitated by mobile genetic elements (MGEs), particularly conjugative plasmids, such as the Tcp plasmids, the Pcp plasmids and the pIP404-like plasmids [2], and insertion sequences (IS) elements. These MGEs mediate horizontal gene transfer as well as contribute to the genomic plasticity and pathogenic adaptability of the bacterium [16]. Increasing evidence suggests that virulence genes often co-localize with MGEs [17,18], enabling rapid acquisition and dissemination under selective pressures.

Pangenome and phylogenomic analysis offer powerful tools for dissecting the genetic diversity, evolutionary dynamics, and functional potential of *C. perfringens* populations from diverse ecological and epidemiological backgrounds [8]. Increasingly, *C. perfringens* is recognized as a pathogen that circulates across multiple interconnected reservoirs rather than being confined to isolated hosts or foodborne events. Livestock production systems, slaughterhouses, and associated processing environments form an ecological continuum in which *C. perfringens* populations can persist, diversify, and exchange genetic material [19]. Strains originating from animals, food products, and environmental matrices may be repeatedly introduced, selected, and redistributed, creating opportunities for the maintenance and spread of virulence- and antimicrobial-resistance-associated traits. The detection of toxigenic and antimicrobial-resistant *C. perfringens* in animal-derived foods, farm environments, and processing facilities underscores the potential for transmission along an integrated animal-environment-food-human continuum, rather than through single-point contamination events alone [17,20]. In this context, genome-resolved approaches that combine core-genome phylogeny with detailed characterization of mobile genetic elements provide a robust framework for disentangling dissemination pathways and for evaluating the public health relevance of *C. perfringens* across interconnected ecological compartments. Several studies involved whole genome sequencing and comparative genomic analysis of *C. perfringens* strains from animal origin [8,21,22], however, isolates from the ecological continuum still is limited. To increase the diversity of sequenced *C. perfringens* and improve our understanding of the pathogenic potential and evolutionary trajectories, whole-genome sequencing and comparative genomic analysis were performed on 33*C. perfringens* isolates derived from animal food production chain. The virulence genes, antimicrobial resistance determinants, and mobile genetic elements, including plasmids, IS elements, and prophages, were profiled, and phylogenetic relationships were inferred from a core-genome maximum-likelihood tree constructed with 119 publicly available genomes. These findings will provide novel insights into the pathogenic potential and evolutionary trajectories of *C. perfringens*, thereby informing the development of effective surveillance and control strategies.

## 2 Material and methods

### 2.1 Bacterial strains

A total of 33*C. perfringens* strains were previously identified in our earlier studies [20,23,24], isolated from samples of cattle, sheep, goat, pork, and poultry-associated food product chains, during various stages of breeding [20], slaughtering [23], milking [20], retailing processing [24] in Shaanxi, Gansu and Jilin provinces, and saved by food safety and public health laboratory of College of Veterinary Medicine of Northwest A&F University (Yangling, Shaanxi, China). The detailed information of all isolates is provided in Table S1.

### 2.2 Sample preparation of isolates and whole-genome sequencing

Each isolate was streaked onto tryptose sulfite cycloserine (TSC, Aobox, Beijing, China) agar plates and incubated in a Bugbox Plus anaerobic workstation (BugBox, Ruskinn Technology Limited, Bridgend, UK) at 37 °C for 24 h to obtain pure single colonies prior to DNA preparation and sequencing. A single typical black colony was inoculated into brain heart infusion broth (BHI, Aobox, Beijing, China) and incubated overnight under anaerobic conditions at 37 °C. Bacterial cells were harvested by centrifugation at 4 °C, 5000 rpm for 10 min, washed twice with sterile distilled water, and then delivered with dry ice to Novogene Co., Ltd. (Beijing, China) for whole-genome sequencing.

### 2.3 Genome assembly, annotation, and typing

Raw reads were quality-filtered to remove adapter sequences and low-quality bases. The filtered reads were de novo assembled using Unicycler 0.5.0 to generate draft genome assemblies [25]. The final contig files in FASTA format produced by Unicycler were used for all downstream analyses unless otherwise specified. Assembly quality was evaluated using CheckM 1.2.0 with the lineage-specific workflow. Annotation was performed using Prokka 1.14.6 and the RAST 2.0 online server (https://rast.nmpdr.org/). To further confirm the taxonomic identity of the sequenced isolates, the assembled genome sequences were analyzed using the Type Strain Genome Server (TYGS, https://tygs.dsmz.de/).

Multi-locus sequence typing (MLST) was conducted using MLST 2.23.0 and the PubMLST database (https://pubmlst.org/) to determine phylogenetic grouping and sequence types (STs). In addition, a core-genome multilocus sequence typing (cgMLST), based phylogeny was constructed to provide higher-resolution genomic relationships among isolates. Comparative topological analysis was conducted to evaluate the concordance between MLST- and cgMLST-based phylogenies by using tanglegrams.

### 2.4 Detection of virulence and antimicrobial resistance genes

Virulence genes were identified using ABRicate 1.0.1 in conjunction with the Virulence Factors Database (http://www.mgc.ac.cn/VFs/). Antimicrobial resistance genes (ARGs) were screened using ResFinder (https://cge.food.dtu.dk/services/ResFinder/).

### 2.5 Identification of Mobile genetic elements

Insertion sequences were identified using BLASTn against the ISfinder database (https://isfinder.biotoul.fr/about.php).

To identify plasmid-associated elements in the *C. perfringens* genomes by performing a combined approach of whole-genome alignment and signature gene screening. Genome assemblies were aligned against three representative plasmids—pCW3 (DQ366035.1), pCP13 (AP003515.1), and pIP404 (NC_001388.1) using BLASTn. In parallel, Abricate 1.0.1 was used to detect plasmid-borne functional genes utilizing a custom-built database derived from the conjugative loci, *tcp* (*tcpA* ∼ *tcpH*) of pCW3-like (*Tcp* locus, e.g., NC_010937.1), and *pcp* (*pcp48* and *pcp49*) loci of pCP13-like (*Pcp* locus, e.g., AP003515.1) plasmids. Isolates carrying ≥3 *Tcp* or *Pcp* locus genes were considered to harbor the corresponding plasmid type, as previously described [26]. Additionally, isolates encoding both *pcpB4* and *pcpD4* were defined as harboring pCP13-like plasmids, in accordance with established molecular markers. A pan-genome analysis was also conducted as a secondary strategy to further confirm plasmid distribution in the isolates.

Prophage regions were predicted using the PHASTER web (https://phaster.ca/), each prophage was categorized into one of 3 confidence levels, intact, questionable, or incomplete. The predicted prophage regions were further extracted and subjected to functional annotation using virulence factor databases to identify potential virulence-associated genes carried by the phage elements. Since some isolates contained ≥1 prophage regions matching the same reference phage, the heatmap displays, for each isolate-phage pair, the maximum match score observed among regions to avoid double-counting, the full information are provided in Table S4.

### 2.6 Pan-genome and phylogenetic analysis

Pan-genome analysis was performed on the 119 public genomes from the NCBI database together with the 33 isolates sequenced in this study using Roary 3.13.0 [27]. A maximum likelihood phylogenetic tree was constructed based on the core genome alignment using IQ-TREE 2.0. Tree visualization was conducted using EvolView (https://evolgenius.info/evolview-v2/). Genomic clustering was performed using the R package fastbaps [28] on the core-genome alignment.

## 3 Results

### 3.1 Genomic information of strains

TYGS-based genome taxonomy analysis confirmed that all 33 sequenced isolates belonged to *C. perfringens*, the whole-genome sequences of which have been deposited in the NCBI database under BioProject accession number: PRJNA1380348. The genome sizes of the isolates ranged from 2,777,327 bp (SM191020–1) to 3,762,689 bp (CF1908105–1) (Table S2). On average, each genome encoded approximately 2900 coding sequences (CDSs) and 73 tRNA genes (Table S2). The GC content varied between 27.0% (WHD-1) and 31.0% (SM191020–1) (Table S2).

Based on the PubMLST framework, the 33 isolates were classified into 16 distinct STs by MLST analysis, indicating substantial genetic diversity within the collection (Table 1). Notably, 7 novel STs were identified in this study, namely from ST1072 to ST1079, expanding the known STs repertoire of *C. perfringens*, among these, ST1077 comprised two Shaanxi-origin isolates (LC20 and JC15). Additionally, 2 Shaanxi isolates (B190311 and BI191201) were assigned to ST282, and 2 Gansu isolates (SD201291–2 and SE191042–2) were assigned to ST370. The remaining STs were each represented by a single isolate, such as ST126 (JT1), ST822 (F2–4), ST876 (LC1) and ST240 (DP18119), further underscoring the high ST diversity among these isolates. Several isolates exhibited incomplete allelic profiles but showed high similarity to existing STs, such as JC2 and LC13 shared 87.5% allelic identity with ST364 and ST437, respectively, JZ10 shared 75.0% allelic identity with ST437, B190497 shared 75.0% allelic identity with ST11, while 3 Jilin isolates (SM191020–1, SM191021–3, and SS191042–2) shared 62.5% allelic identity with ST78.

**Table 1:** Characteristics of identified sequence types of *C. perfringens.*

Strain	Region	Toxinotype	ST
B190311	Shaanxi	A	282
B190335	Shaanxi	A	1072*
B190447	Shaanxi	A	1073*
B190497	Shaanxi	D	11 (75.0%)
BA1903151	Shaanxi	A	1074*
BI191201	Shaanxi	A	282
BT190286	Shaanxi	A	34 (62.5%)
F2–4	Shaanxi	A	822
JC15	Shaanxi	A	1077*
JC2	Shaanxi	A	364 (87.5%)
JT1	Shaanxi	A	126
JZ10	Shaanxi	A	437 (75.0%)
CF1908105–1	Shaanxi	A	485 (50.0%)
WHD-1	Shaanxi	G	381 (50.0%)
DA18121	Shaanxi	A	17 (37.5%)
DH18118	Shaanxi	A	1075*
DM19087–1	Shaanxi	A	290 (37.5%)
DP18119	Shaanxi	A	240
DP190532	Shaanxi	A	1076*
E3D	Gansu	D	936
LC1	Shaanxi	A	876
LC13	Shaanxi	A	437 (87.5%)
LC20	Shaanxi	A	1077*
SD1901	Jilin	A	337
SD201291–2	Gansu	A	370
SD201293–1	Gansu	A	123 (50.0%)
SE191042–2	Gansu	A	370
SE201235–1	Gansu	A	1078*
SF201263–1	Gansu	A	1079*
SFS19102–2	Jilin	A	69 (25.0%)
M191020–1	Jilin	A	78 (62.5%)
SM191021–3	Jilin	F	78 (62.5%)
SS191042–2	Jilin	F	78 (62.5%)

Note: The table summarizes strain origin, toxinotype and multilocus sequence typing (MLST) assignments. Newly identified sequence types are marked with an asterisk (*). Partially matched STs indicate the percentage allelic identity to the closest PubMLST reference profile.

### 3.2 Comparative topological congruence between MLST and cgMLST

**Fig. 1:**
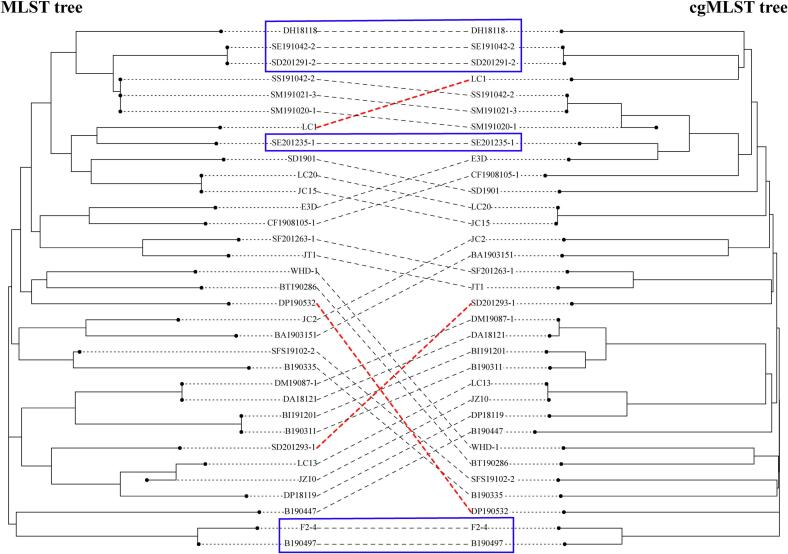
Comparison of MLST- and cgMLST-based phylogenetic relationships among 33 isolates. The left tree represents the phylogeny inferred from seven-locus MLST, and the right tree represents the phylogeny based on core-genome MLST. The same isolates in the two trees are connected by dashed lines. Blue boxes indicate isolates showing consistent or largely similar positions between the MLST and cgMLST trees. Red dashed lines highlight isolates with markedly different positions between the two trees, indicating discordant placement between MLST and cgMLST. (For interpretation of the references to colour in this figure legend, the reader is referred to the web version of this article.)

A tanglegram was constructed to compare MLST- and cgMLST-based trees with emphasis on isolate clustering and relative placement rather than branch lengths (Fig. 1), the two phylogenies exhibited mostly topological congruence, with both conserved and discordant isolate placements evident across the dataset.

Strict topological concordance was observed for 6/33 isolates marked blue box in Fig. 1, including DH18118, SE191042–2, SD201291–2, SE201235–1, F2–4, and B190497, showed identical relative positions in both trees, indicating that their MLST assignments accurately reflected underlying core-genome relationships. In addition, 24/33 isolates displayed partially concordant placement, characterized by parallel but oblique linkages between the two trees. This pattern suggests that, while MLST captured broad lineage affiliation, it lacked the resolution to fully resolve fine-scale genomic divergence. By contrast, pronounced topological discordance was evident for LC1 (ST876), DP190532 (ST1076), and SD201293–1 (ST123 with 50.0% allelic identity). These isolates occupied markedly different positions in the two trees, with multiple crossover linkages, indicating unstable correspondence between MLST-based grouping and genome-wide relatedness. Notably, these isolates were associated with either novel STs or incomplete allelic profiles, suggesting that allelic convergence or missing loci may obscure true chromosomal relationships.

Collectively, these results demonstrate that seven-locus MLST provides a useful first-level framework for population structuring but may group genomically divergent strains into the same or closely related STs. In contrast, cgMLST offers higher discriminatory power and more accurately resolves fine-scale chromosomal relationships, particularly among isolates derived from heterogeneous hosts and production environments.

### 3.3 Virulence genes and ARGs profiling

**Fig. 2:**
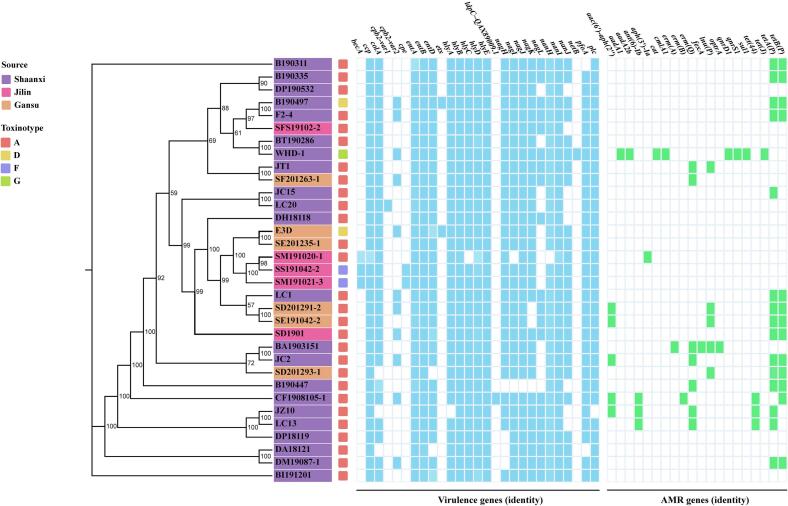
Core-genome phylogeny and distribution of virulence and antimicrobial resistance genes among 33 isolates. The core-genome phylogenetic tree was constructed based on the Roary-derived core gene alignment using IQ-TREE. Bootstrap values (>50%) are shown at branch nodes. Isolates are colour-coded according to geographic source and toxinotype, as indicated in the left panels. The presence or absence of virulence genes (blue) and antimicrobial resistance genes (green) is shown as a heatmap aligned with the phylogeny. Gene identity thresholds were applied as described in the Methods. The integrated visualization highlights the conserved distribution of core virulence genes across phylogenetic lineages, as well as the heterogeneous and lineage-independent patterns of toxinotype-defining loci and antimicrobial resistance determinants. (For interpretation of the references to colour in this figure legend, the reader is referred to the web version of this article.)

The distribution of virulence genes and ARGs profiling in the 33 *C. perfringens* genomes was examined (Fig. 2). A total of 27 virulence-associated genes were identified, with individual genomes encoding between 14 (B190447) and 22 loci (B190497, CF1908105–1 and WHD-1) (Fig. 2). When mapped onto the phylogenetic tree, virulence genes showed a clear dichotomy between toxinotype-defining markers and broadly conserved core virulence factors.

Toxinotype-specific genes were strictly lineage-restricted. The enterotoxin gene *cpe* was detected exclusively in the 2 type F isolates (SM191021–3 and SS191042–2), *etx* occurred only in the 2 type D isolates (B190497 and E3D), and *netB* was confined to the type G isolate WHD-1. These genes were localized to discrete terminal branches of the tree, consistent with stable maintenance within specific toxinotypes.

In contrast, a core virulence repertoire was widely conserved across phylogenetic lineages and isolation sources. Genes encoding α-toxin (*plc*), perfringolysin O (*pfoA*), collagenase (*colA*), sialidases (*nanH*, *nanI* and *nanJ*), and GH84 hyaluronidases (*nagI*-*nagK*) were present in more than 90% of genomes, forming a shared virulence backbone among livestock-associated isolates (Fig. 2). Additional hemolysin genes (*hlyB*, *hlyD*, *hlyE*), cysteine protease (*ccp*), and enterotoxin-associated genes (*entA* and *entB*) were universally detected, whereas *cpb2* displayed allelic variation, with the atypical variant (*cpb2-var2*) present in 11 isolates and the consensus variant (*cpb2-var1*) restricted to a single strain (LC20).

ARG profiling revealed that 21 of the 33 isolates (54.5%) carried at least one ARG, with 20 distinct ARGs identified spanning eight antimicrobial classes (Fig. 2), eight isolates (24.2%) met a multidrug-resistance profile which carrying ARGs resistant to at least three antimicrobial classes. Tetracycline resistance genes were the predominant, with *tetA(P)* and *tetB(P)* detected in 15 (45.5%) and 13 (39.4%) isolates, respectively, while the less common genes, *tet(44)* is found in LC13, CF1908105–1 and JZ10 (9.1%), and *tet(J)* in WHD-1 (3.0%), respectively. Macrolide resistance was mainly associated with *erm(Q)*, present in 7 isolates (21.2%), whereas *erm(A)* and *erm(B)* were only detected in a single strain BA1903151 and CF1908105–1, respectively. Among aminoglycoside-modifying enzymes, *aac(6′)-aph(2″)* was detected in 15.2% isolates (5/33), *ant(6)-Ib* in 9.1% isolates (JZ10, CF1908105–1 and LC13), and *aph(3′)-Ia* was uniquely present in SM191020–1. The genes *aadA1* and *aadA2b* were detected only in WHD-1. Fluoroquinolone resistance genes *qnrS1* and *qnrD1* were likewise uniquely found in WHD-1, which also carried *sul1*. Chloramphenicol resistance genes were confined to WHD-1 (*cat*, *cmlA1*). Lincosamide and oxazolidinone resistance genes were identified only in BA1903151, which harbored both *lnu(P)* and *optrA*.

At the isolate level, ARG carriage ranged from 1 to 8 genes (63.6%), 12 isolates (36.4%) lacked detectable ARGs. Three isolates (9.1%, JC15, SF201263–1 and SM191020–1) carried a single ARG, 8 isolates (24.2%) carried 2 ARGs, and 2 isolates (6.1%, B190447, SD201293–1) carried 3 ARGs. Four isolates (12.1%, JC2, LC13 SD201291–2 and SE191042–2) harbored 4 distinct ARGs. Larger ARG complements were observed in JZ10, CF1908105–1 and BA1903151, each carrying 5 genes (9.1%). WHD-1 exhibited the most extensive resistance pattern, harboring 8 ARGs and representing a high-burden multidrug-resistant genotype.

### 3.4 Analysis of Mobile genetic elements

#### 3.4.1 Plasmid distribution revealed by a dual strategy based on BLAST and Abricate

To identify the three major *C. perfringens* plasmid families (pCW3-like, pCP13-like, and pIP404-like), reference-based BLASTn screening and Abricate detection of hallmark genes (*tcpA-H*, *pcp48*/*pcp49*, *bcn5*) were jointly used under standard thresholds.

**Fig. 3:**
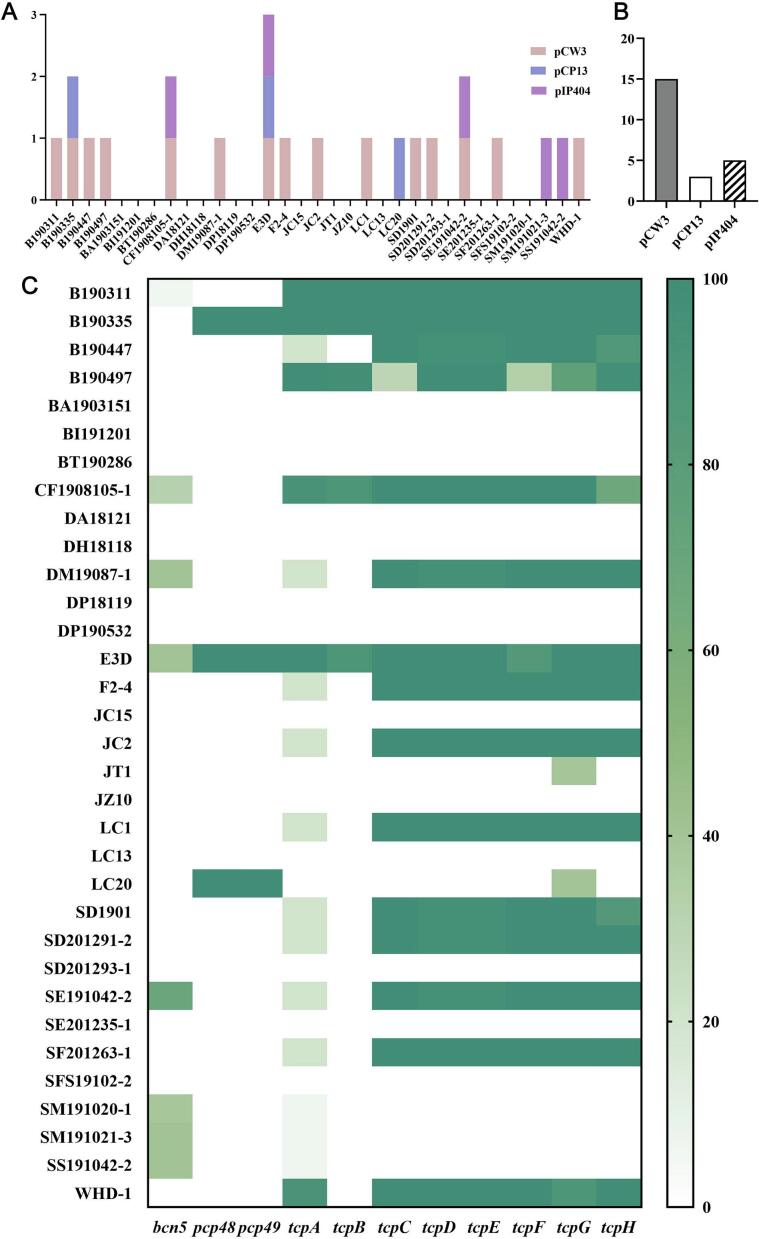
Distribution of pCW3-like, pCP13-like, and pIP404-like plasmids inferred from BLAST and Abricate analysis. A Radial representation of plasmid family distribution among the isolates based on BLASTn comparisons with reference plasmids pCW3 (DQ366035.1), pCP13 (AP003515.1), and pIP404 (NC_001388.1) (≥50% coverage, ≥70% nucleotide identity). B Number of isolates carrying each plasmid type: 15 (45.5%) harbored *pCW3*-like, 3 (9.1%) *pCP13*-like, and 5 (15.2%) *pIP404*-like plasmids. C Heatmap of conjugative *tcp* (*tcpA*–*tcpH)* and *pcp* (*pcp48*, *pcp49*) loci screened by Abricate. Most *pCW3*-positive isolates contained multiple *tcp* genes with high identity, while all *pCP13*-positive isolates carried both *pcp48* and *pcp49*. *pIP404*-like plasmids were detected solely by BLASTn due to the absence of conjugative modules.

BLASTn analysis showed that 18 of the 33 *C. perfringens* isolates (54.5%) carried at least one plasmid (Fig. 3A), with 15 isolates (45.5%) harboring pCW3-like plasmids, 3 isolates (B190335, E3D, and LC20, 9.1%) carrying pCP13-like plasmids, and 5 isolates (CF1908105–1, SE191042–2, SM191021–3, SS191042–2, and E3D, 15.2%) containing pIP404-like plasmids (Fig. 3A, B). No plasmid sequences were detected in the remaining 15 genomes.

Abricate-based screening of conjugative loci confirmed that all pCW3-like plasmid positive isolates contained at least 3 *tcp* genes, consistent with the BLASTn results. All three pCP13-like isolates carried *pcp48* and *pcp49* with 100% identity, whereas no *pcp* genes were detected in the remaining strains (Fig. 3C). The identification of pIP404-like plasmids only relied on BLASTn alignments since it lacks canonical conjugation machinery. Notably, B190335 exhibiting the highest sequence coverage. Co-occurrence of different plasmid families was observed in 4 isolates (B190335, CF1908105–1, E3D and SE191042–2), most prominently in E3D, which harbored all 3 plasmid types (Fig. 3A).

#### 3.4.2 Diversity and distribution of IS elements

A comprehensive identification and comparative analysis of ISs from the genomes of the isolates revealed a total of 81 distinct IS types. A total of 31 IS types were identified, together with Tn-type transposons family. The ISs number of each genome varied markedly, ranging from 2 (WHD-1) to 44 elements (BA1903151) (Table S3). Among all identified ISs, the types *ISCpe4* was the highest prevalent ISs which detected in 100% (33/33) isolates (Table S3). Additional IS types with high detection frequencies included *ISCpe2* (13/33, 39.4%), *IS1469* (12/33, 36.4%), *ISCpe3* (12/33, 36.4%), *IS1470* (11/33, 33.3%), *ISCbt3* (10/33, 30.3%) and *ISClsp5* (10/33, 30.3%) (Table S3). Two transposon-related elements Tn2 and Tn3, were both detected in isolate SM191020–1 with 100% sequence identity. Notably, WGS assemblies enabled locus-scale comparison of the *cpe*-containing region, which were similar in size (∼19 kb), while an round full-length *IS1151*-like element was detected on the same *cpe*-containing region in the 2 type F isolates, which the *cpe* localization had been validated in our previous study based on PCR detection and spore heat-resistance profiling, that SM191021–3 as chromosomal *cpe* (*c-cpe*) and SS191042–2 as plasmid-borne *cpe* (*p-cpe*) [29].

#### 3.4.3 Prophage distribution and Association of Virulence Genes

**Fig. 4:**
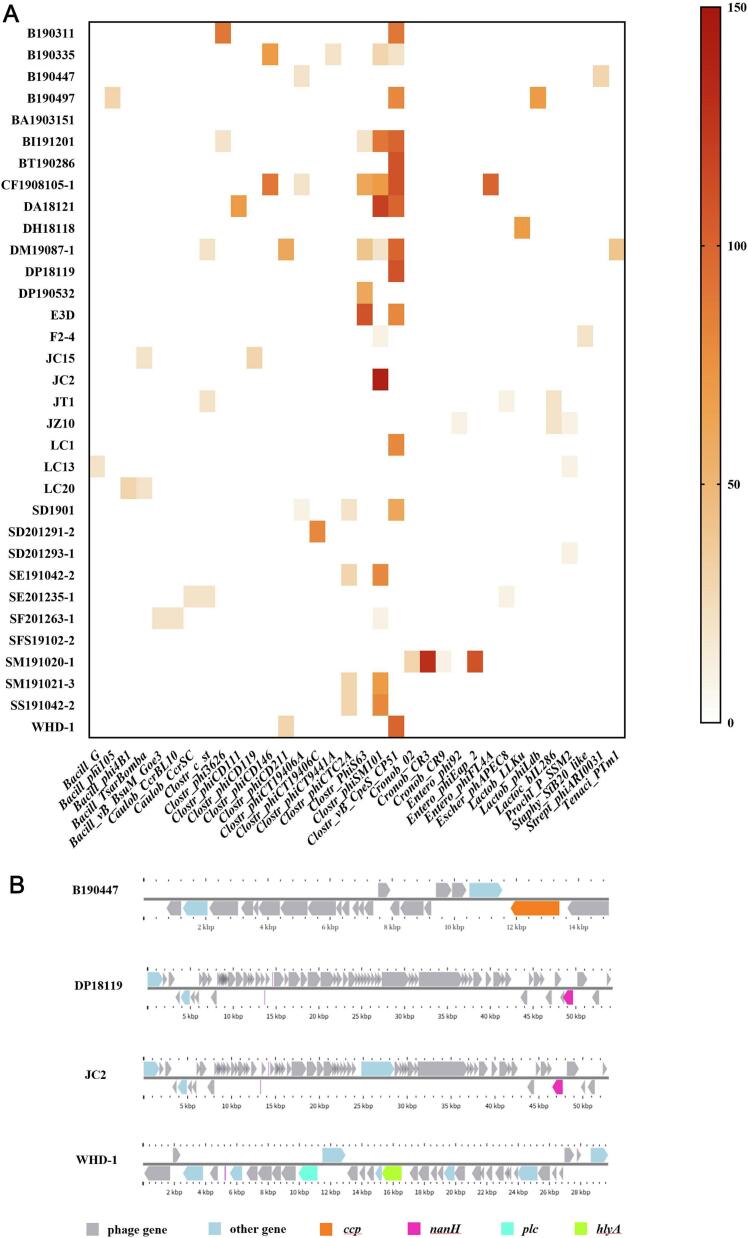
Prophage diversity and prophage-encoded virulence factors in isolates. This figure illustrates the prophage composition identified within *C. perfringens* genomes and the virulence factors carried by these prophages. A Heatmap showing the presence and distribution of prophage regions identified by PHASTER in all isolates. Each block represents a prophage hit, with the colour gradient indicating sequence identity. A total of 81 prophage regions corresponding to 34 unique phage types were detected, categorized as intact, questionable, or incomplete based on gene content and structural integrity. B Genome maps of prophage regions containing virulence genes, illustrating the genetic organization and localization of *ccp* (orange), *nanH* (pink), *plc* (bright blue), and *hlyA* (green) within prophage genomes. (For interpretation of the references to colour in this figure legend, the reader is referred to the web version of this article.)

PHASTER analysis identified 81 prophage regions in the 33 genomes, representing 34 distinct phage types. These regions were classified as intact (*n* = 13), questionable (*n* = 20), or incomplete (*n* = 48) based on genomic organization and structural completeness. The 31 isolates (93.9%) carried at least one prophage region, the number of prophage regions per genome ranged from 1 (21.2%) to 7 (6.1%) (Table S4), with most isolates carrying 1 (21.2%) or 2 (39.4%) prophages. Six isolates (18.2%) contained ≥4 prophage regions, including B190335, BI191201, CF1908105–1, DA18121, DM19087–1, and SM191020–1, showing a high level of prophage load. In particular, CF1908105–1 and DM19087–1 carried multiple prophages of distinct types, exhibiting the diverse phage integration repertoire in their genomes (Fig. 4A). The predicted prophage regions ranged from 5.6 kb to 54.2 kb, encoded 6 to 61 proteins, and exhibited GC contents of 26% ∼ 30% for most isolates (Table S4). Among the predicted regions, *Entero_phiEap*, *Cronob_CR3* and *Cronob_CR9* within SM191020–1 displayed unusually high GC contents (>45%), demonstrating marked compositional divergence from the host genome. *Clostr_vB_CpeS_CP51*, detected in 13 isolates (39.4%), followed by *phiSM101* in 11 isolates (33.3%).

Functional annotation identified four virulence-associated genes—*ccp*, *nanH*, *plc*, and *hlyA*, were located within prophage regions of several isolates (Table 2). Sequence comparison showed high nucleotide conservation (92.6–99.6% identity, 95–100% coverage). Specifically, *ccp* in B190447 shared 97.1% identity with *Clostridium phiCT19406A*, *nanH* in DP18119 exhibited 92.6% identity to *Clostr_vB_CpeS_CP51*, whereas *nanH* in JC2 showed 92.8% identity to phiSM101. In WHD-1, *plc* and *hlyA* were located within the *Clostr_phiCD211* prophage and showed >99% sequence identity. No virulence genes were detected in prophage regions of the remaining 27 isolates (Fig. 4B).

**Table 2:** Virulence-associated genes identified within prophage regions of 4 isolates.

Strain	Phage	Gene	Identity (%)
B190447	*Clostr_phiCT19406A*	*ccp*	97.13
DP18119	*Clostr_vB_CpeS_CP51*	*nanH*	92.62
JC2	*Clostr_phiSM101*	*nanH*	92.76
WHD-1	*Clostr_phiCD211*	*plc*	99.58
		*hlyA*	99.13

Note: The table lists prophage types, embedded virulence genes, and corresponding nucleotide identities relative to reference phage sequences.

### 3.5 Pangenome and phylogenetic analysis

**Fig. 5:**
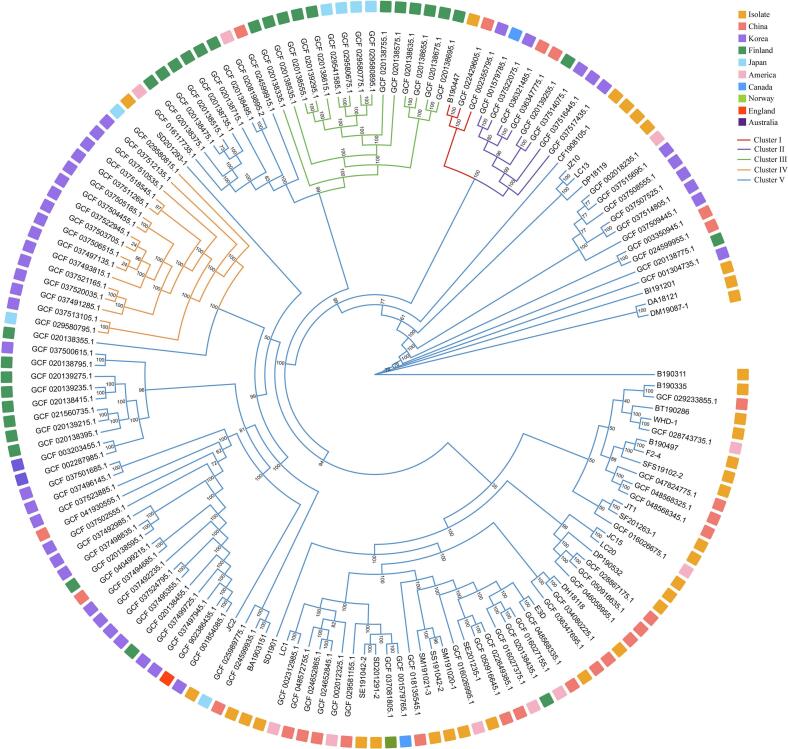
Core-genome maximum-likelihood phylogeny of *C. perfringens* isolates and reference genomes. Five major chromosomal clusters (Clusters I-V) were resolved, and branch colours correspond to these phylogenetic clusters, highlighting their evolutionary separation. Genomes sequenced in this study are marked in orange on the outer ring, while reference genomes are colour-coded by country of origin to illustrate geographic distribution (China, Korea, Finland, Japan, America, Canada, Norway, England, and Australia). Bootstrap support values ≥80% are indicated at internal nodes. The tree shows that most livestock-associated isolates fall within the globally widespread Cluster V, whereas only a single isolate from this study belongs to the less common Cluster I.

The phylogenetic relationships among *C. perfringens* strains were characterized by inferring a maximum-likelihood tree from the core-genome alignment of 33 isolates and 119 publicly available genomes, which showed that the 33 isolates are distributed among multiple well-supported branches interspersed among the reference genomes. Using fastbaps applied to the same core-genome alignment, 152 genomes were classified into 5 genomic clusters. Cluster V represented the predominant lineage (110/152 genomes), encompassing 97.0% (32/33) isolates together with reference genomes from Asia, Europe and North America, consistent with a widely distributed, globally mixed lineage. Only isolate, B190447, originating from cattle, was located in the small Cluster I together with 2 reference genomes from a yak in China (GCF_022429605.1) and the human gut in Korea (GCF_002355795.1), forming a distinct, sparsely represented lineage with 100% bootstrap support. Cluster II consisted of a limited set of reference genomes from Canada, Finland, China and Korea, whereas Cluster III comprised almost exclusively Finnish and Japanese reference genomes and Cluster IV grouped mainly Japanese and Korean reference genomes (Fig. 5).

Within Cluster V, the Chinese livestock-associated isolates were further subdivided into several well-supported sublineages that often were consistent with their ecological origin. At one end of the tree, B190311 (cattle sample) and BI191201 (cattle intestines) each formed an individual long external branch, adjacent to a third branch containing two dairy-associated isolates, DA18121 (milking-parlor air) and DM19087–1 (raw milk). Together, these 3 branches represent a set of closely related cattle- and dairy-chain lineages that nevertheless exhibit substantial chromosomal divergence from the main body of Cluster V. CF1908105–1, originating from cattle feed, formed a distinct single-isolate branch located closest to a compact subclade comprising JZ10 (chicken feet), LC13 (sausage), and DP18119 (tools and personnel in a dairy farm), with all corresponding nodes supported by 100% bootstrap values (Fig. 5). It indicates that several food- and environment-derived isolates form a coherent lineage with modest chromosomal divergence despite their heterogeneous production-chain origins.

A second major sublineage within Cluster V comprised a broad assemblage of isolates from diverse livestock-production matrices. SF201263–1 (goat feed) and JT1 (chicken leg), B190335 (cattle sample) and BT190286 (slaughterhouse tools), together with the poultry-associated isolates WHD-1 (type G, chicken feces) and F2–4 (chicken feces), the goat-derived isolate SFS19102–2 (goat feces), and the cattle-derived B190497 (type D), clustered tightly with 4 Chinese and 2 U.S. reference genomes (100% bootstrap support).

Adjacent to this group, a larger and more diffuse clade contained LC20 (sausage), JC15 (chicken wing), DP190532 (tools and personnel from a dairy farm), SD1901 (sika deer large intestine), DH18118 (bovine body surface swab), together with 7 additional field isolates and multiple reference genomes from China, U.S., Canada, Norway, and Finland (>80% bootstrap support). Within this clade, the 2 type F isolates, SM191021–3 (mutton) and SS191042–2 (sheep fleece swab), formed a well-supported subbranch with SM191020–1 (mutton), which clustered most closely with a Chinese bovine-derived reference genome (GCF_018135545.1) and the United States clinical reference genome (GCF_016026995.1). This sublineage also included the type D strain E3D, which grouped most closely with reference genomes from China (GCF_048568335.1), U.S. (GCF_016027375.1 and GCF_016027155.1), and Finland (GCF_020138435.1) (Fig. 5).

## 4 Discussion

*C. perfringens* is a major pathogen at the human-animal-environment interface, and resolving its genomic diversity is essential for surveillance of virulence and antimicrobial-resistance determinants. In this study, 33 isolates were obtained from the beef processing of slaughter house [23], and the breeding and milking process of dairy farms [20] from 2018 to 2020 in three provinces of China, as well as chicken meat and cured meat products such as sausage in 2013 reported in our previous studies [24]. This extensive sources, spanning animal hosts, slaughterhouse and milking environments and animal derived products, provides a region-specific view of *C. perfringens* circulating along cattle-, sheep-, goat-, pork-, and poultry-associated food product chains, and supports previous observations that food animals and their production environments represent important reservoirs for this species [8,20,23,24,30,31].

The genomes sequenced in this study fell within the expected size and GC range of *C. perfringens*, confirming the conserved low-GC and compact chromosomal architecture typical of the species [19,32]. This background aligns with reports that *C. perfringens* has an open pangenome and frequently acquires accessory genes via horizontal gene transfer [33], a process proposed to be facilitated by low GC content [8,32]. Integration of MLST, cgMLST and core-genome phylogenetic analyses provided complementary insights into the population structure of the 33 isolates. Although MLST classified the collection into 16 sequence types, including 7 newly identified STs (Table 1), comparison with cgMLST and the core-genome phylogeny revealed only partial concordance between ST assignment and chromosomal relatedness. This indicates that identical STs unnecessarily correspond to tightly clustered lineages at the core-genome level. Clear concordance was observed for some host-matched isolates. The bovine-derived strains B190311 and BI191201, both assigned to ST282, clustered adjacently in the cgMLST tree and occupied neighboring branches within Cluster V of the core-genome phylogeny, supporting ST282 as a stable lineage in this dataset. In contrast, other STs encompassed greater chromosomal heterogeneity. Within the ST1077 lineage, the two isolates LC20 and JC15 showed close placement in both the MLST and cgMLST frameworks, indicating concordant clustering between seven-locus typing and core-genome phylogeny for this sequence type. Conversely, isolates with only partial MLST matches, such as the ST78-related Jilin isolates (SM191020–1, SM191021–3 and SS191042–2), formed a well-supported subbranch in the core-genome phylogeny, suggesting a shared chromosomal backbone that was not fully captured by MLST. Placement of the isolates within a global phylogenetic framework further clarified these patterns. Thirty-two of the 33 isolates were embedded within Cluster V, the predominant lineage comprising reference genomes from Asia, Europe and North America, consistent with a geographically widespread and globally mixed chromosomal background. A similar pattern showed substantial genomic heterogeneity and lacked strict clustering by host or geographic origin [22]. In contrast, B190447 was assigned to the small and sparsely represented Cluster I together with two East Asian reference genomes, indicating that less common chromosomal lineages also circulate in livestock-associated environments. Together, these observations demonstrate that while MLST remains a useful first-pass tool for cataloguing diversity, higher-resolution genome-based approaches are essential to resolve fine-scale relatedness and to distinguish true lineage continuity from allelic convergence within dominant global *C. perfringens* backgrounds [19,34].

*C. perfringens* exerts its pathogenic effects mainly through the secretion of toxins and degradative enzymes rather than active invasion of host cells, and more than 20 exotoxins and enzymes have been implicated in tissue damage and enteric disease in humans and animals [2,35]. Among the isolates, a conserved set of virulence-associated genes contrasted with the heterogeneous distribution of toxinotype-defining loci. The conserved backbone including *plc*, *pfoA*, *colA*, the sialidases *nanH*/*nanI*/*nanJ*, and the GH84 hyaluronidases *nagI*-*nagK*, presented in nearly all isolates (90.9–100%), indicating a shared baseline capacity for mucosal colonization and tissue invasion among livestock-associated ecological niches. This pattern is consistent with previous studies showing that *C. perfringens* usually carries a conserved set of toxin- and enzyme-related genes, whereas the *cpb2* is present only in a smaller proportion of strains [8]. Notably, recent studies reported that *pfoA* is frequently absent from a human-derived hypovirulent lineage and suggested that lower *pfoA* prevalence may reflect lineage-specific attenuation or niche adaptation [5,26]. In contrast, *pfoA* was highly prevalent in our animal-derived collection, consistent with differences in lineage composition and sampling source between livestock-associated and certain human-associated populations. Overall, these data support a model in which a conserved virulence backbone persists across diverse hosts and phylogenetic backgrounds, while toxinotype-defining loci show greater variability and more clearly track specific sources and lineages.

The *netB* carrier WHD-1, recovered from a chicken with necrotic enteritis, is in line with the established role of NetB in lesion development and disease pathogenesis [36], is also consistent with the close association of *netB* with avian-origin isolates [21]. WHD-1 was isolated from broiler chickens suffering necrotic enteritis in 2013, predated the national prohibition of antibiotic growth promoters in feed. The ARGs profile is consistent with long-term use of antimicrobial growth promoters and therapeutic antibiotics in broiler production prior to regulatory restrictions, which has been linked to the accumulation of multidrug-resistance loci in poultry-associated *C. perfringens* and other enteric bacteria [37,38]. Taken together with our phenotypic data, these findings point to poultry production and enclosed processing environments as key hotspots for the selection and dissemination of multi-resistant *C. perfringens* lineages.

The β2-toxin gene *cpb2* was detected mainly in isolates (33.3%) from animal derived products (Fig. 2), with the atypical *cpb2-var2* allele predominating, in agreement with reports that atypical *cpb2* is common among ruminant and beef-processing isolates [23,39]. Interestingly, *cpb2* was not detected in the 2 *cpe* + isolates in this study, despite prior reports that β2-toxin may be involved in antibiotic-associated diarrhea (AAD) and sporadic diarrhea (SD) in some *cpe*-positive backgrounds [40,41]. In contrast, the consensus *cpb2-var1* allele was detected only in the pork sausage-derived isolate LC20, suggesting that the 2 *cpb2* variants may differ in host or ecological associations rather than representing a simple presence-absence trait, and also it is not a reliable marker of enteric disease [42].

An important insight from our dataset is that type F isolates from the ovine chain share an IS-linked *cpe* module despite different *cpe* localization backgrounds. *Clostridium perfringens* enterotoxin (CPE), encoded by *cpe*, is the primary toxin responsible for gastrointestinal symptoms in both foodborne and non-foodborne human illness [43], chromosomal *cpe* (*c-cpe*) are predominantly associated with food poisoning, plasmid *cpe* (*p-cpe*) is more frequently linked to non-food-borne GI, such as AAD and SD [44]. Interestingly, marker-based plasmid profiling suggested a pIP404-like, but not pCW3-like, signature in both type F isolates. At the locus level, their *cpe*-associated regions lacked the canonical IS elements typically described in well-characterized *cpe* loci, such as *IS1469*/I*S1470* in chromosomal *cpe* contexts or *IS1470*-like sequences in some plasmid-borne *cpe* loci, both carried a near full-length *IS1151*-like element within the *cpe* genetic context, supporting an IS-linked architecture for the enterotoxin locus [45]. Both isolates also carried *becA*, a marker typically associated with pCP13-like plasmids [46], highlighting that multiple toxin-related mobile elements may co-circulate in ovine-chain type F backgrounds. The co-occurrence of *cpe*, *IS1151*-like, and *becA* in these 2 isolates points to a conserved, potentially transmissible virulence configuration, although long-read sequencing will be required to determine whether these loci are physically co-localized on the same replicon and to clarify their evolutionary origin. Importantly, recovery of these type F isolates from mutton and a sheep fleece swab demonstrates that enterotoxigenic lineages are already present along the ovine meat production and processing chain, underscoring the need for targeted monitoring and hygiene interventions in small-ruminant slaughter and processing facilities.

In general, *p-cpe* isolates usually carry *nanI*, typically together with *nanH* and *nanJ*, whereas *c-cpe* food-poisoning strains almost always lack *nanI* and instead carry *nanH* alone or *nanH* + *nanJ* [47]. As known, NanI and NanJ are secreted sialidases, while NanH is largely intracellular during vegetative growth but can be released unless mother-cell lysis during sporulation [48,49]. In our collection, however, both the *c-cpe* isolate and the *p-cpe* isolate encoded the complete *nanH*/*nanI*/*nanJ* set, indicating that the putative sialidase-associated colonization/nutrient-scavenging repertoire can be maintained independently of *cpe* localization. Given this unexpected genotype, we further examined whether the sialidase genes were differentially expressed between the two type F isolates. Both strains were cultured under identical conditions, and total RNA was extracted at 12 h followed by cDNA synthesis. The transcriptional levels of *nanH*, *nanI* and *nanJ* were quantified by RT-qPCR using 16S rRNA as the internal control. Notably, all three sialidase genes showed significantly higher expression in SM191021–3 than in SS191042–2. Among them, *nanH* exhibited a highly significant difference (*p* < 0.01), whereas *nanI* and *nanJ* showed significant differences (*p* < 0.05). Based on the pronounced transcriptional divergence of *nanH*, we subsequently constructed *nanH* deletion mutants in both isolates to further investigate the role of NanH in regulating CPE production and secretion.

Transposition-related elements have been documented in *C. perfringens*, including the chloramphenicol resistance transposons *Tn4451*/*Tn4452* and the *cpe*-associated transposon-like element *Tn5565*, highlighting the contribution of transposition to genome plasticity in this species [50]. In our dataset, Tn2 and Tn3 were identified in the type A isolate SM191020–1, despite the absence of pCW3-, pCP13-, and pIP404-like plasmid signatures. This finding suggests that transposition modules may persist in alternative genetic contexts and could contribute to strain-specific genome remodeling independent of the three major plasmid families.

Overall, the ARG profiles in our animal-derived collection were consistent with patterns reported for livestock-associated *C. perfringens*, with tetracycline determinants (*tetA(P)*/*tetB(P)*) and macrolides (*erm(Q))* remaining the dominant genes (Fig. 2), similar to trends described in human-derived cohorts [26] and consistent with historical tetracycline use in food-animal production [51] as well as reports from clinical and farm-associated settings [52]. Additional macrolide, lincosamide, aminoglycoside and fluoroquinolone resistance genes were detected only sporadically, likely reflecting heterogeneous antimicrobial exposures across production environments [53]. Importantly, BA1903151, which carried *optrA* together with *fexA*, *erm(A)*, *erm(Q)*, *lnu(P)* (Fig. 2, Fig. 3A), to our knowledge, this is the first report of an *optrA*-positive *C. perfringens* recovered from slaughterhouse air. Since the first description in *Enterococcus faecalis* E349 in 2015 [54], *optrA* has been detected in diverse Gram-positive and Gram-negative species from humans, hospital effluents, foods and food animals, and in a sheep-derived *C. perfringens* strain QHY-2 where *optrA*-*fexA*-*erm* genes reside on a pCW3-like plasmid [15]. Because *optrA* mediates resistance to linezolid and tedizolid, the recovery of an *optrA*-positive isolate from slaughterhouse air is noteworthy. Several pCW3-positive isolates (B190311, B190497, DM19087–1, LC1, SD1901) (Fig. 3A) mainly harbored tetracycline genes (e.g., *tetA(P)*/*tetB(P)* (Fig. 2), especially, pCW3-like plasmid was carried in WHD-1 which harboring 8 ARGs exhibited multiple resistance uniquely (Fig. 2), consistent with previous reports that key ARGs (e.g., *erm(Q)*, *tetA(P)* and *optrA*) can be associated with pCW3-like/pCP13-like plasmids [15,55]. However, isolates (BA1903151 and JZ10) carried multiple ARGs but lacking plasmid markers, E3D with pCW3/pCP13/pIP404 markers present but no ARG detected, these patterns suggest that ARGs in our isolates likely reside in diverse genetic contexts (e.g., non-typed plasmids, chromosomal insertions, or other MGEs) and may disseminate via multiple transmission routes.

Mobile genetic elements played central but distinct roles in shaping accessory-genome diversity in current study. pCW3-like conjugative plasmids showed a strong association with the atypical *cpb2-var2* allele, among the 15 isolates assigned as pCW3-like, *cpb2-var2* was detected in 11 strains (73.3%), in line with previous studies demonstrating that *tcp*-positive pCW3-like plasmids are major vehicles for *cpb2* and other toxin genes in *C. perfringens* [18,55]. Additionally, LC20 with a clear pCP13-like signature, which carried 19 toxin genes including *cpb2-var1* allele, *becA* and *colA*, in agreement with the previous studies reported that consensus *cpb2* frequently resides on pCP13-family toxin plasmids together with additional virulence factors such as the binary enterotoxin *bec* and collagen-binding adhesins [56]. The core *tcp* locus is typically defined as an 11-gene module (*intP* and *tcpA*-*tcpJ*), and mutational analysis have shown that *tcpA*, *tcpF* and *tcpH* are critical for efficient transmechanistic basis for the broad dissemination of these plasmid-borne traits [57]. Composite plasmid complements were less common (Fig. 3), illustrating that individual *C. perfringens* strains can stably carry several distinct toxin- and bacteriocin-encoding plasmids simultaneously.

Prophage analysis supplemented a further layer of variation, 4 isolates harbored intact prophage regions encoding virulence-associated genes. In particular, the *phiCD211* region in WHD-1 (Fig. 4) that carried 2 virulence-associated loci. *phiCD211*-like is pointed to a lysogenic backbone, was described contributing to strain diversity, and its genome carries multiple integrase and other recombination-associated genes, hallmarks of temperate phages capable of chromosomal integration and excision [58], WHD-1 carried 22 virulence genes supported it once again. In DP18119, the largest prophage region corresponded to *Clostr_vB_CpeS-CP51* (*ΦCP51*) and contained an annotated virulence-associated locus. *ΦCP51* has been described previously as an inducible prophage (e.g., by mitomycin C), supporting stable lysogeny with the capacity for SOS-linked induction [59]. Notably, *phiSM101* has been described as an episomal phage of the enterotoxigenic *C. perfringens* strain SM101 and encodes the endolysin Psm with potent lytic activity against *C. perfringens* [60]. *PhiSM101* prophage regions were detected in 11 isolates, including 9 type A strains (*cpe*-negative) and 2 type F strains. The presence of *phiSM101*-like modules across multiple toxinotypes suggests that these phage elements can circulate beyond classical enterotoxigenic lineages, potentially through modular exchange among clostridial phages. Overall, our findings align with the view that *C. perfringens* prophages can contribute to toxin mobility and virulence evolution [61,62]. Although prophage activity cannot be inferred from sequence data alone, the localization of virulence loci within prophage regions supports the hypothesis that phage-associated regulatory circuitry and induction dynamics may influence virulence expression [63].

For example, prophage-rich genomes CF1908105–1 and DM19087–1 carried 7 prophage regions, CF1908105–1 harbored 27 IS copies, carried both pCW3-like and pIP404-like plasmid markers, as well as five ARGs and 22 virulence genes. By contrast, DM19087–1 contained 5 IS copies, carried only the pCW3, 2 ARGs, and 20 virulence genes. BA1903151 carried no prophage regions and no detectable plasmid markers, contained the highest IS copies (44), 5 ARGs and 19 virulence genes. In this study, *ISCpe4* was detected in all isolates (33/33), indicating that this element is a highly conserved and widespread component of the *C. perfringens* mobilome. *ISCpe4* belongs to the *IS200*/*IS605* family and is assigned to the *IS1341* group. Members of the *IS200*/*IS605* family are characterized by a distinctive single-strand “peel-and-paste” transposition mechanism mediated by the small TnpA transposase, which can promote localized genome remodeling and generate insertion hotspots without requiring classical DDE cut-and-paste chemistry [64].

Importantly, both type F isolates lacked acquired ARGs (Fig. 2), indicating that enterotoxigenic potential and antimicrobial resistance were not obligatorily coupled in this dataset. Given the limited number of type F genomes analyzed here and the absence of comparable AMR data from other *cpe*-positive strains in the literature, it cannot be determined whether the presence or genomic location of *cpe* is associated with ARG burden. Larger datasets of type F isolates from diverse sources systems will be required to clarify potential links between enterotoxin genotype and antimicrobial resistance profiles. Taken together, the lineage-resolved integration of phylogeny and accessory profiles highlights distinct epidemiological signals along modern animal-derived food chains. Collectively, these results support the value of lineage-resolved genomic surveillance that jointly tracks the conserved chromosomal scaffold and the dynamic mobile-element landscape to inform targeted control strategies for this important zoonotic pathogen. Synthesis of lineage structure and the mobilome. Integrating core-genome phylogeny with virulence, AMR and MGE profiles indicates that a relatively conserved chromosomal scaffold underpins livestock-associated *C. perfringens*, whereas plasmids, prophages and IS elements generate strain-level functional divergence on this background. Within the dominant Cluster V lineage, multiple toxinotypes occurred within the same chromosomal sublineages, consistent with toxin loci being mobile and capable of reshaping toxinotype without wholesale chromosomal change [65]. Across strains, ARG burden, prophage content and IS copy number varied semi-independently of typed plasmid carriage, supporting a multi-component mobilome in which different elements contribute complementary routes for accessory-gene turnover. The ubiquitous detection of *ISCpe4* (33/33) further suggests a shared IS-rich backbone in production-chain isolates that may provide recurrent substrates for microevolutionary change [64]. This study has several limitations. The number of isolates analyzed remains modest and geographically restricted, and the use of short-read sequencing limits definitive resolution of plasmid and prophage architectures as well as precise localization of virulence and resistance determinants. These constraints restrict the ability to fully reconstruct transmission pathways and to distinguish between chromosomal and mobile-element-mediated dissemination across interconnected animal, food, and environmental reservoirs. Future studies integrating long-read sequencing with expanded sampling across hosts, production systems, food products, and environmental matrices will be essential to resolve mobile genetic modules, quantify rates of gene gain and loss, and better define the dynamics of strain persistence and spread. Together, such lineage-resolved genomic surveillance approaches will provide a stronger evidence base for risk assessment and targeted intervention strategies aimed at mitigating the circulation of *C. perfringens* across linked ecological compartments.

## Funding

None.

## Ethics approval and consent to participate

Not applicable.

## Consent for publication

The manuscript has been approved by all authors for publication.

## CRediT authorship contribution statement

**Zhaoyu Chang:** Writing – review & editing, Writing – original draft, Visualization, Software, Methodology, Formal analysis, Data curation, Conceptualization. **Haoyu Zhao:** Writing – review & editing, Software, Methodology, Formal analysis, Data curation. **Xinru Wang:** Formal analysis, Data curation. **Yue Dong:** Formal analysis, Data curation. **Juan Wang:** Software, Methodology, Formal analysis. **Deyang Guo:** Data curation. **Kailiang Han:** Visualization, Data curation. **Yanfen Jiang:** Writing – review & editing, Writing – original draft, Data curation, Conceptualization.

## Declaration of generative AI and AI-assisted technologies in the writing process

During the preparation of this work the authors used ChatGPT version 5.2 to improve readability and language. After using this tool/service, the authors reviewed and edited the content as needed and take full responsibility for the content of the published article.

## Declaration of competing interest

The authors declare that there is no conflict of interests regarding the publication of this paper.

## Data Availability

The data that has been used is confidential.

## Appendix A Supplementary data

Supplementary material Image 1
